# Adjuvant chemotherapy for rectal cancer with complete pathological response (pCR) may not be necessary: a pooled analysis of 5491 patients

**DOI:** 10.1186/s12935-019-0851-9

**Published:** 2019-05-14

**Authors:** Xiang Hu, Ya-Qi Li, Xiao-ji Ma, Long Zhang, San-Jun Cai, Jun-Jie Peng

**Affiliations:** 1 Department of Colorectal Surgery, Fudan University Shanghai Cancer Center, 270 Dong’an Road, Shanghai, 20032 China; 2 Department of Oncology, Shanghai Medical College, Fudan University, Shanghai, 200032 China

**Keywords:** Rectal cancer, Complete response, Adjuvant chemotherapy, Neo-adjuvant chemoradiotherapy, Survival

## Abstract

**Background:**

It is recommended postoperative adjuvant chemotherapy for all rectal cancers undergoing neo-chemoradiotherapy regardless of the final yield pathology. However, the role of adjuvant chemotherapy in pathological complete response (pCR) remains controversial. We aimed to identify the necessarily of adjuvant chemotherapy in pCR.

**Methods:**

Consecutive patients with pCR in Fudan University Shanghai Cancer Center (FUSCC) were enrolled. Meanwhile, a pooled analysis of individual patient with pCR was performed from PubMed and Embase databases for validation.

**Results:**

A total of 171 patients form FUSCC were identified to achieve pCR with up to almost 10 years follow-up. Among them, those receiving adjuvant chemotherapy had no survival benefits compared to those without adjuvant chemotherapy (log-rank test = 0.17, P = 0.676). The 5y-DFS rates for patients in chemo group and no-chemo group was 87.5 and 88.8%, respectively, showing no significant difference (p = 0.854). No matter chemotherapy regimens, T stage, EMVI and CRM status varied, the results remained consistent. Meantime, the COX model did not demonstrate adjuvant chemotherapy as the independent risk factor for OS and DFS. Additionally, among 18 systemic recurrences in all, the rate of relapse surged rapidly on the 12 months and rose up to peak in the 36th months. In order to validate these results, nine controlled trials involving 5491 patients with pCR were included in this pooled-analysis. For both 5-year overall survival and disease-free survival, the pooling data did not produce a statistically significant effect in cases of adjuvant chemotherapy performed (RR = 0.79 and RR = 0.95, respectively, all p > 0.05).

**Conclusion:**

This study suggested that rectal cancer patients with pCR did not benefit from adjuvant chemotherapy and we recommended that achievement of pCR require more prolonged close follow care in case of distant metastasis.

## Background

Neo-adjuvant chemoradiotherapy (CRT) followed by radical surgery is the recommended optimal treatment for locally advanced rectal cancer [1], as the preoperative chemoradiotherapy could reduce recurrence and improve survival [2]. Similarly, post-operative adjuvant chemotherapy has an established role in patients with locally advanced rectal cancer for reducing the risk of recurrence and mortality up to 20–30% [3]. Then, is adjuvant chemotherapy necessary to the patients with complete response (pCR)? As once pCR has achieved, it will be followed with very low local recurrence, distant metastasis and improved long-term survival [4]. According to the present guidelines, adjuvant chemotherapy is offered as a standard treatment for all patients receiving CRT and the radical surgery, regardless of the postoperative pathological results. The NCCN recommended postoperative adjuvant chemotherapy for all patients undergoing preoperative chemoradiotherapy regardless of the final yield pathology, even pCR [5], while, the ESMO guidelines stated that “similar to the situation in colon cancer Stages III (and “high-risk” Stage II), adjuvant chemotherapy can be provided, even if the scientific support for sufficient effect is less” [6]. However, the various expert panels regarded definition of high risk differed slightly, generally including T4 tumor, poor differentiation, inadequate lymph node retrieval, the circumferential resection margin(CRM), extramural venous invasion (EMVI). Hence, no consensus was arrived on the basis of pCR in determining the necessity for adjuvant chemotherapy. Despite the widespread use of chemotherapy, solid evidence to support the benefits of adjuvant chemotherapy after pCR and radical excision is lacking [7]. What is more, several authors have demonstrated that pCR patients cannot benefit from the adjuvant chemotherapy after CRT and the curative resection [8–10], however their studies showed characteristic of small size sample and selection bias. With these heterogeneous results and potential harmful effects about chemotherapy, we determined to assess the value of adjuvant chemotherapy after achievement of pCR following preoperative radiology and surgery in rectal cancer patients.

## Materials and methods

### Data source

Since 2006, all patients undergoing surgery for colorectal cancer at the Department of colorectal Surgery, Fudan University Shanghai Cancer Center (FUSCC), Shanghai, China, are scheduled for periodic follow-up at our cancer center. All patient data are prospectively entered in the FUSCC database, including age, race, tumor location, year of diagnosis, primary tumor size, histological grade, number of lymph nodes examined, type of radiation, marital status, preoperative multimodal treatment, details of the surgical procedure, occurrence of complications, postoperative histopathology, application of adjuvant therapy, and follow-up (date of last visit, tumor recurrence, date of tumor-related or unrelated death, overall and disease-free survival). The complete response was defined as ypT0N0M0 (absence of invasive cancer and in situ cancer in the rectum, reginal nodes and no imaging metastasis). The clinical staging prior operation was based on imaging studies including high resolution rectal magnetic resonance (MRI) and abdominopelvic contrast-enhanced computed tomography (CT) scan. Neo-adjuvant chemoradiotherapy regimen included a total of 50 Gy radiation (2 Gy per day for 25 fractions over 5 weeks) with concurrent chemotherapy of CapeOX or single-agent Capecitabine regimen. All patients from FUSCC dataset have provided written informed consent. The research protocol was reviewed and approved by the institutional review board of the FUSCC. Once the pCR was achieved, adjuvant chemotherapy or not will be based on the will of patients.

### Search strategy for systematic analysis

A literature search was carried out in the electronic databases including PubMed, Web of Science, EMBASE and the Cochrane Central Register of Controlled Trials on February 1st, 2019. We employed the key words “rectal cancer, rectal carcinoma, neoplasms” and “chemotherapy, adjuvant” and “chemoradiotherapy, chemoradiotherapy, chemoradiation, radiotherapy” and “complete response, pCR, ypCR” with limits of “Clinical Trial” and “Humans”. The related article references were also enriched the search. All available randomized controlled trials (RCTs) and comparative cohort studies evaluating the efficacy of adjuvant chemotherapy after pCR with at least one quantitative outcome were enrolled. The primary outcome was 3 or 5-year OS, and the 3 or 5-year DFS. Two authors independently extracted the below data: authors, publication year, study design, age, adjuvant chemo (schedules), median follow-up, OS, and DFS. Any disagreement was resolved by a senior author. When several studies by the same database reported the same outcomes at similar follow-up periods, we included in our analysis either the better quality or the most informative publication.

### Inclusion and exclusion criterion

All clinical controlled trials, evaluating the value of adjuvant chemotherapy after achievement of pCR following neo-adjuvant chemoradiotherapy and surgery in rectal cancer patients, were selected for eligible. Trials were enrolled without limitation to nations, year, or language. We excluded studies in which the outcomes of interest were not reported, or it was impossible to calculate these from the published results. No control group, of course, was within our exclusion criteria.

The quality of included randomized trials were assessed as three categories from A (high quality) to C (low quality). These quality criteria included the randomization procedure, the use of intention-to-treat analysis, the report of dropout rates, allocation concealment and the extent to which valid outcomes were described. But this analysis included all case–control studies, which quality were not high.

### Statistical analysis

Statistical evaluation was performed using IBM SPSS statistics Version 22 (SPSS Inc; IBM Corporation Software Group, Somers, NY). The Chi square test or Fisher exact test was utilized for exploratory comparisons of patient groups. All statistical tests were performed 2-sided, and p values less than 0.05 were considered to be statistically significant. Observed (unadjusted overall) survival was estimated with the Kaplan–Meier method, and the log-rank (Mantel-Cox) test was used to compare independent subgroups. Overall survival and disease-free survival were used as the primary outcome parameter. Overall survival may represent disease-specific survival regarding the initial malignant disease and considers only tumor related deaths as events in this study [11]. Cox proportional hazard models were used to investigate the effect on survival of multivariable relationships among covariates including the age at diagnosis, gender, stage at diagnosis, EMVI, CRM and treatment. Stage, or any known clinical characteristics supposed to affect the prognosis were the stratified variable. Hazard ratios (HRs) and 95% confidence intervals (CIs) for multivariate analyses were computed using the Cox proportional hazards regression models.

This systematic comparison was carried out in line with Cochrane Collaboration recommendations of meta-analyses guidelines [12]. For categorical variables, the relative risk (RR) as the summary statistics was employed, demonstrating the adverse ratio in the study group (chemo group) relative to control group (no-chemo group). A relative risk of less than one was the favor of the study group, and the point estimate of the relative risk was taken of statistical significance at the p = 0.05 level, if the 95% confidence interval did not include the value 1. A fixed effects model was used on the presumption that variation in the individual trial results occurred about a true mean. Conversely, the randomized model was adopted. All statistical analyses were carried out with Stata 10 (StataCorp, College Station, Tex).

## Results

### Patient characteristics and tumor presentation in FUSCC

From the 950 patients with neo-adjuvant chemoradiotherapy and curative resection in the FUSCC colorectal cancer dataset from 2006 to 2016, we focused the rectal cancer patients with pCR with up to 10-year follow-up (11–138 months). There was a total of 171 patients with under peritoneal reflection who achieved pCR. Of the inclusion patients, 115 patients received adjuvant chemotherapy (Chemo group) and others did not receive therapy (No-chemo group) got close hospital follow-up care. The detailed comparison of concerning characteristics of Chemo group and No-chemo group was listed in Table 1. Of not, the pair-wise comparisons of all covariates were not significant with p more than 0.05 (Table 1), which demonstrated the characteristics were balanced between two treatments groups. Above all, the preoperational high-risk parameters also did not show any difference between the two groups, just like the circumferential resection margin(CRM), extramural venous invasion (EMVI).

**Table 1:** Clinical and demographics characteristics of patients with pCR, stratified by adjuvant chemotherapy in the FUSCC

Variables	No chemo (56)	chemotherapy (115)	p value
Gender			
Female	27(48.2)	37(32.2%)	0.62
Male	29(51.8)	78(67.8)	
Age, years	57.6 ± 11	54.5 ± 10.8	0.085
Clinical T stage			0.145
T2	28(50.0)	71(61.7)	
T3/4	28(50.0)	44(38.3)	
Clinical N sage			0.724
N0	17(30.4)	38(33.0)	
N1–2	39(69.6)	77(67.0)	
Clinical TNM staging			0.724
II	17(30.4)	38(33.0)	
III	39(69.6)	77(67.0)	
CRM			0.296
Negative	33(58.9)	58(50.4)	
Positive	23(41.1)	57(49.6)	
EMVI			0.823
Negative	39(69.6)	82(71.3)	
Positive	17(30.4)	33(28.7)	
Operation			0.058
AR	25(44.6)	69(60.0)	
APR	31(55.4)	46(40.0)	
Lymph node examined			0.844
Median	10	9	
Adjuvant chemotherapy regimen			
Cape	–	53	
CapeOX		62	

CRM, circumferential resection margin; EMVI, extramural venous invasion; AR, anterior resection; APR, abdominoperineal resection

### Overall survival

**Fig. 1:**
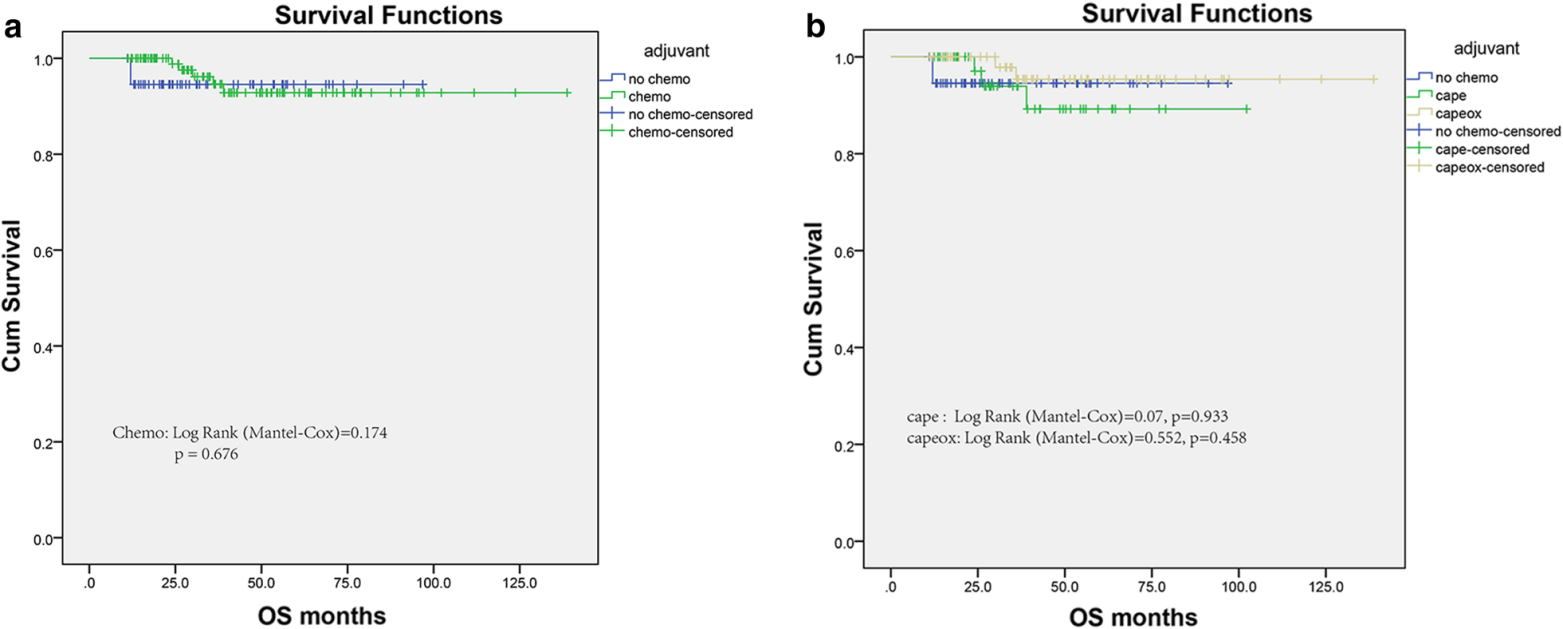
Kaplan-Meier survival plots of overall survival (**a**) based on the receipt of adjuvant chemotherapy in the FUSCC. **b** stratified by the varied chemotherapy regimens provided in the Chemo group, Cape group (Mono-Chemotherapy with Capecitabine) and Capeox group (combination chemotherapy with Capecitabine and Oxaliplatin)

Kaplan–Meier survival analysis did not reveal a significant difference in OS between those undergoing adjuvant chemotherapy or not. (log-rank test = 0.17, p = 0.676) (Fig. 1a). And the per-protocol analysis demonstrated an HR of 0.737 for overall survivals (95% CI 0.176–3.093) for adjuvant chemotherapy. Multivariable Cox analysis also confirmed that adjuvant chemotherapy did not provide additional survival profit after achievement of pCR. In detail, among the patients receiving adjuvant chemotherapy, 94.6% were survived at the end of the follow-up period time, comparing to 94.5% of patients who did not receive chemo treatment. The 10-year cumulative overall survival was estimated comparable as 94.5% and 92.8% in those who received adjuvant chemotherapy or not. Additionally, the cumulative rectal overall survival was presented in the same pattern at 1, 3, 5, and 10-year regarding to adjuvant chemotherapy (Table 4). In light of the varied chemotherapy regimens provided in the Chemo group, we further explored the outcomes among three patient cohorts: No chemo group, Cape group (Mono-Chemotherapy with Capecitabine) and Capeox group (combination chemotherapy with Capecitabine and Oxaliplatin). Consistently, not a significant difference was found in OS between the No chemo group and two chemotherapy regimens cohorts (Fig. 1b). Using conventional general logistic regression to test for interaction analyses, no interactions with any of the baseline characteristics were statistically significant with the adjuvant chemotherapy (P > 0,05).

**Fig. 2:**
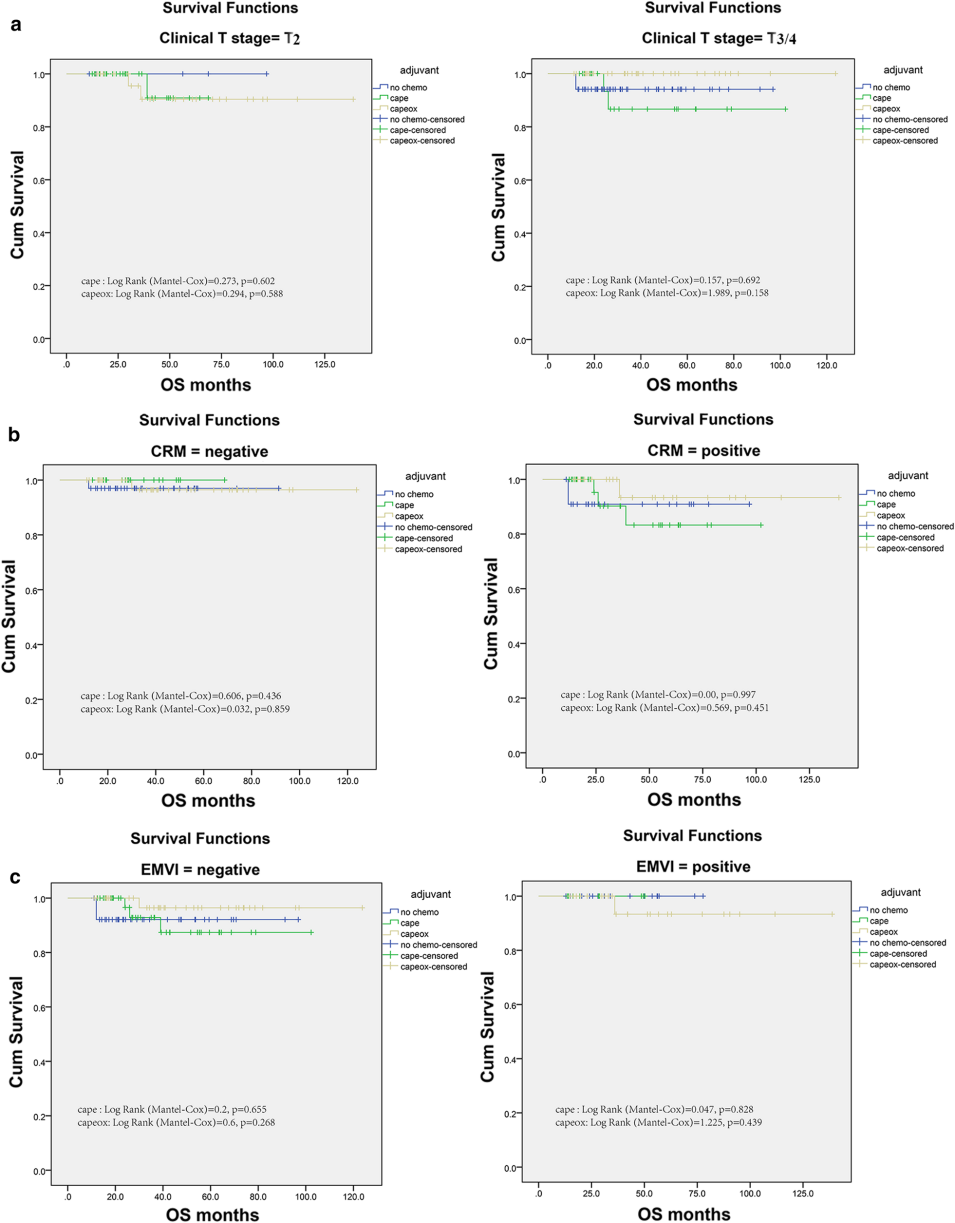
Impact of T stage (**a**), EMVI (**b**) and CRM (**c**) on the overall survival among the No-chemo group, Cape group and Capeox group

Then we stratified patients according to the clinical covariates, and identified the impact of adjuvant chemotherapy on survival in different subgroups. In first, based on the T stage, in both stage T2 patients and T stage 3/4, the overall survival of adjuvant chemotherapy was comparable with that in the no chemo group, no matter Mono or Combined Chemotherapy was administered (Fig. 2a). The multivariable Cox model also did not show apparent OS benefits for chemotherapy in various T stage groups (HR: 0.939, 95% CI 0.234–3.747), see Table 2. Above all, extramural venous invasion (EMVI) and circumferential resection margin (CRM) detected by high-resolution magnetic resonance imaging have been historically regarded as prognostic factors for rectal cancers, which significantly widened the opportunities to identify individual patient risk and adapted the treatment plan accordingly. In line with stage-subgroups, adjuvant chemotherapy did not have significant roles in subgroups of EMVI and CRM. Chemotherapy seemed not improve survival in patients with positive or negative EMVI and CRM (Fig. 2b, c). Furthermore, we performed Cox proportional model and got the same results: the HR offered by EMVI and CRM as (HR: 0.316; 95% CI 0.039–2.568) and (HR: 3.539; 95% CI 0.714–17.539) respectively, see Table 2.

**Table 2:** Multivariate analyses of prognostic factors for overall survival in the FUSCC

Variables	Cox proportional hazards		
	Hazard ratio	95% CI	p value
Adjuvant chemotherapy			0.678
No	1 (reference)		
Yes	0. 739	0.176–3.093	
Gender			0.019
Female	1 (reference)		
Male	0.081	0.010–0.658	
Age	1.058	0.985–1.135	0.12
Clinical T stage			0.926
T2	1 (reference)		
T3/4	0. 937	0.234–3.747	
Clinical N sage			0.697
N0	1 (reference)		
N1–2	0.753	0.180–3.150	
CRM			0.122
Negative	1 (reference)		
Positive	3.539	0.714–17.539	
EMVI			0.281
Negative	1 (reference)		
Positive	0.316	0.039–2.568	
Operation			0.361
AR	1 (reference)		
APR	1.949	0.466–8.159	
Lymph node examined			0.064
≤ 12	1 (reference)		
> 12	0.854	0.722–1.009	

CRM indicates circumferential resection margin; EMVI, extramural venous invasion; AR, anterior resection; APR, abdominoperineal resection

### Disease-free survival

**Fig. 3:**
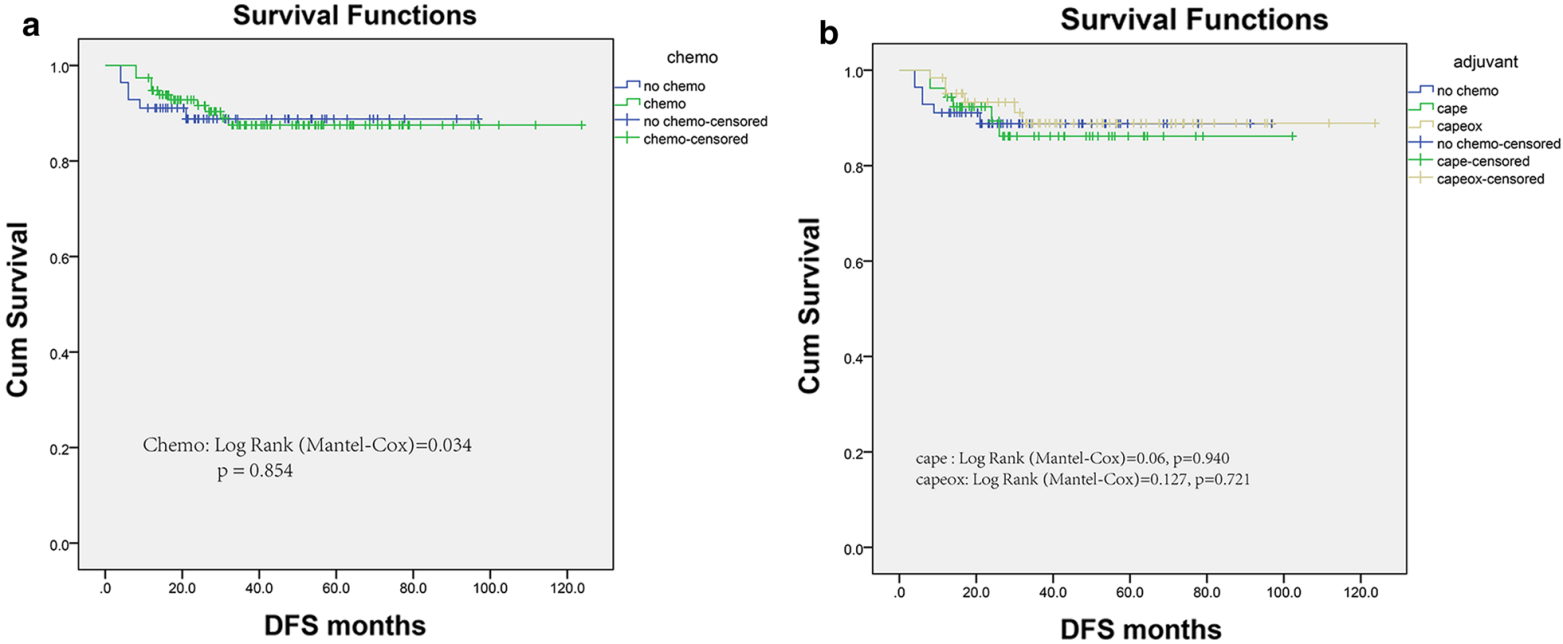
Kaplan-Meier survival plots of disease-free survival (**a**) based on the receipt of adjuvant chemotherapy in the FUSCC. **b** stratified by the varied chemotherapy regimens provided in the Chemo group, Cape group and Capeox group

Kaplan–Meier curves for disease-free survival were presented in Fig. 3a. The prognosis of Chemo group, with 5-year disease-free survival of 87.5%, was comparable with the No chemo group, with 5-year disease-free survival of 88.8% (p = 0.854). what is more, Fig. 3b depicted the disease-free survival curves for colorectal patients with pCR estimated by 10-year span of follow-up. Difference in survival were not found in patients with No chemo group, Mono and Combined Chemotherapy. And the per-protocol analysis demonstrated an HR of 0.912 for disease-free survival (95% CI 0.342–2.432) for adjuvant chemotherapy. In addition, even combined regimen chemotherapy did not provide survival profit for pCR confirmed by multivariable Cox analysis, see Table 3.

**Table 3:** Multivariate analyses of prognostic factors for cancer disease-free survival in the FUSCC

Variables	Cox proportional hazards		
	Hazard ratio	95% CI	p value
Adjuvant chemotherapy			0.855
No	1 (reference)		
Yes	0.912	0.342–2.432	
Gender			0.215
Female	1 (reference)		
Male	1.340	0.844–2.127	
Age	1.016	0.972–1.061	0.481
Clinical T stage			0.446
T2	1 (reference)		
T3/4	0.697	0.275–1.766	
Clinical N sage			0.401
N0	1 (reference)		
N1–2	0.657	0.247–1.751	
CRM			0.707
Negative	1 (reference)		
Positive	1.194	0.474–3.010	
EMVI			0.222
Negative	1 (reference)		
Positive	0. 462	0.134–1.597	
Operation			0.391
AR	1 (reference)		
APR	1.503	0.593–3.809	
Lymph node examined			0.381
≤ 12	1 (reference)		
> 12	0.406	0.054–3.049	

CRM, circumferential resection margin; EMVI, extramural venous invasion; AR, anterior resection; APR, abdominoperineal resection

**Fig. 4:**
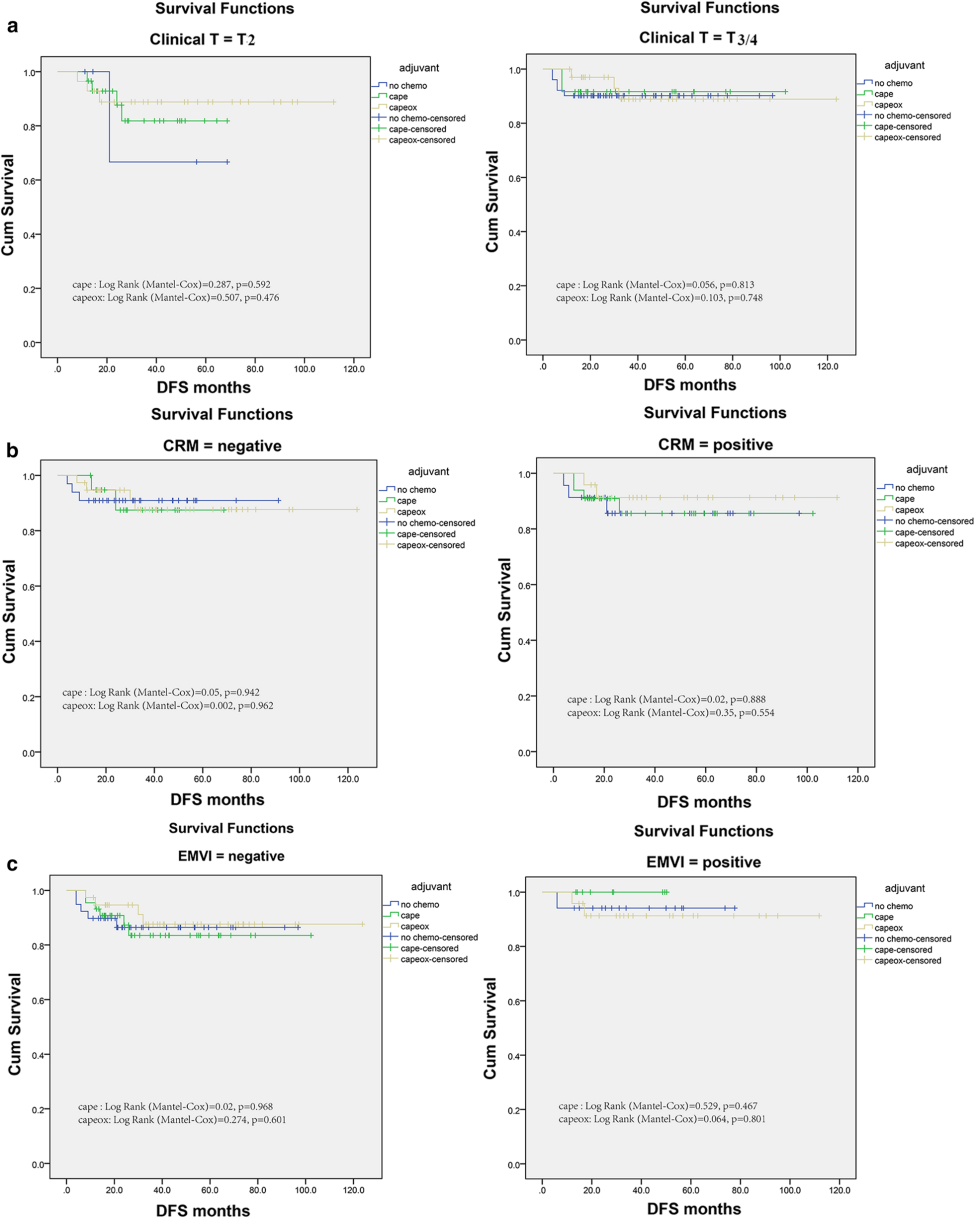
Impact of T stage (**a**), EMVI (**b**) and CRM (**c**) on the disease-free survival among the No-chemo group, Cape group and Capeox group

To identify the chemotherapy related prognostic factor linking DFS, Kaplan–Meier adjusted survival analysis stratified by T stage, EMVI and CRM were used to compare disease-free survival among No chemo group, Cape adjuvant chemotherapy and Capeox chemotherapy group. Similarly, there were no difference in disease-free survival with respect to positive for EMVI and CRM or not, no matter Mono or Combined Chemotherapy was administered (Fig. 4a–c). At the meantime, the results of the Cox proportional hazards analyses for factors associated with disease-free survival were summarized in Table 4. T stage (multivariate Cox HR, 0.697; 95% CI 0.275–1.766; p = 0.446), EMVI (multivariate Cox HR, 0.462; 95% CI 0.134–1.597; p = 0.222), and the status of CRM (multivariate Cox HR, 1.194; 95% CI 0.474–3.010; p = 0.707) were not significantly associated with disease-free survival.

**Table 4:** Estimated year survival rates (1, 3, 5, and 10) in patients with pCR, stratified by adjuvant chemotherapy in the FUSCC

Survival	No chemo	Chemotherapy
Overall survival (patients at risk)		
1-year rate	94.5 ± 3.1 (56)	98.8 ± 1.2 (115)
3-year rate	94.5 ± 3.1 (22)	94.6 ± 1.2 (53)
5-year rate	94.5 ± 3.1 (7)	92.8 ± 3.1 (22)
10-year rate	94.5 ± 3.1 (1)	92.8 ± 3.1 (1)
Disease-free survival (patients at risk)		
1-year rate	91.1 ± 3.8 (56)	93.8 ± 2.3 (115)
3-year rate	88.8 ± 4.3 (20)	92.8 ± 2.5 (49)
5-year rate	88.8 ± 4.3 (6)	87.5 ± 3.5 (21)
10-year rate	88.8 ± 4.3 (0)	87.5 ± 3.5 (0)

### Recurrences

**Fig. 5:**
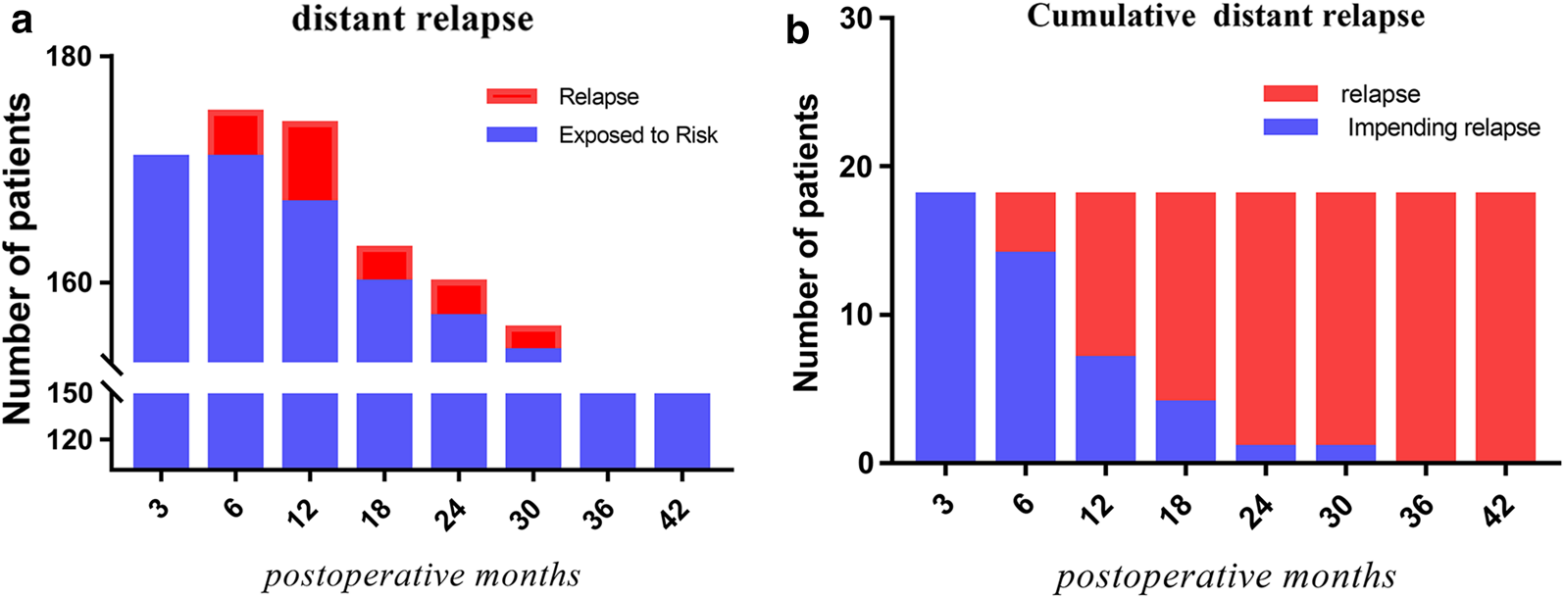
**a** Number of distant relapse in pCR in relation to time at the end of radical surgery. **b** Cumulative numbers of distant relapse relative to the all relapse with the post-operation months

In total, there were 18 distant recurrences, with the most common sites being lung and liver, and no local recurrences occurred in the two groups. At 5 years, the cumulative incidence for distant recurrences was 12.5% in the chemotherapy group and 11.2% in the no chemotherapy group (HR 0.912, 95% CI 0.342–2.432). Similar results were found in per-protocol analysis (HR 0.854, 95% CI 0.71–1.36; p = 0.41). Their cumulative incidence of relapse in terms of time interval post- surgery (Fig. 5a) revealed that the rate of relapse surged rapidly on the 12 months and rose up to peak 10.5% in the 36th month. The percentage of relapse in the interval after surgery was 22.2, 38.9, and 16.6% in the 6th, 12th, and 18th months, respectively (Fig. 5b).

### Systematic comparison of adjuvant chemotherapy after achievement of pCR

In all, 96 related studies were identified from the PubMed and Embase search. After a one by one screening process for the titles and abstracts, 8 trials were selected for the final analysis. As a result, a total of 5491 patients with pCR was enrolled after a literature search performed in the electronic databases [8, 9, 13–18] (Table 5). Then we performed a systematic comparison of the available data to assess the efficacy of adjuvant chemotherapy for pCR treated with neoadjuvant treatment and radical surgery, using 5-year survival (OS and DFS) as endpoints.

**Table 5:** Overview of baseline characteristics per included study

Author	Shahab D	Zhou	Geva R	Gamaleldin M	Kuan FC	Kiran RP	Hu X	CAPIRCI C	Dossa F
Year	2017	2016	2014	2017	2017	2012	2019	2008	2018
Country	USA	China	Israel	USA	China	USA	China	Italy	Canada
Study design	Retrospective	Case control	Case control	Prospective control	Case control	Case control	Case control	Prospective sub-group	Retrospective
N	789/2102	19/21	35/17	47/83	114/145	14/34	115/56	127/439	667/667
Age (c/n)	57.2/61.2	54/54.1	60.9/68.7	55.9/60.6	56.7/61.8	50.5/55.5	54.5/57.6	62	56/57
Gender (male%)	56.8/61.2	36.8/76.2	68.5/47	57.4/67.5	59.6/66.2	50/73.5	67.8/57.8	67	56.8/55.5
cT-stage									
T1–2	110/348	2/2	1/2	NA	11/21	1/4	57/23	29	60/59
T3–4	679/1754	17/19	31/14	NA	102/124	13/30	58/33	409	585/584
cN-stage									
N0	388/1224	6/7	23/12	25/48	33/52	9/22	66/31	NA	319/319
N+	401/878	10/12	9/3	22/34	81/91	5/12	49/25	169	348/348
Surgery									
AR	565/1474	16/12	26/10	5/41	78/108	12/22	69/25	339	NA
APR	183/511	3/9	9/7	42/13	15/16	2/12	46/31	100	NA
Chemotherapy regimen	NA	Combined and single-agent	NA	NA	NA	NA	Combined and single-agent	Combined and single-agent	NA

c/n, chemotherapy group vs. no-chemo group; NA, no available; AR, anterior resection; APR, abdominoperineal resection

**Fig. 6:**
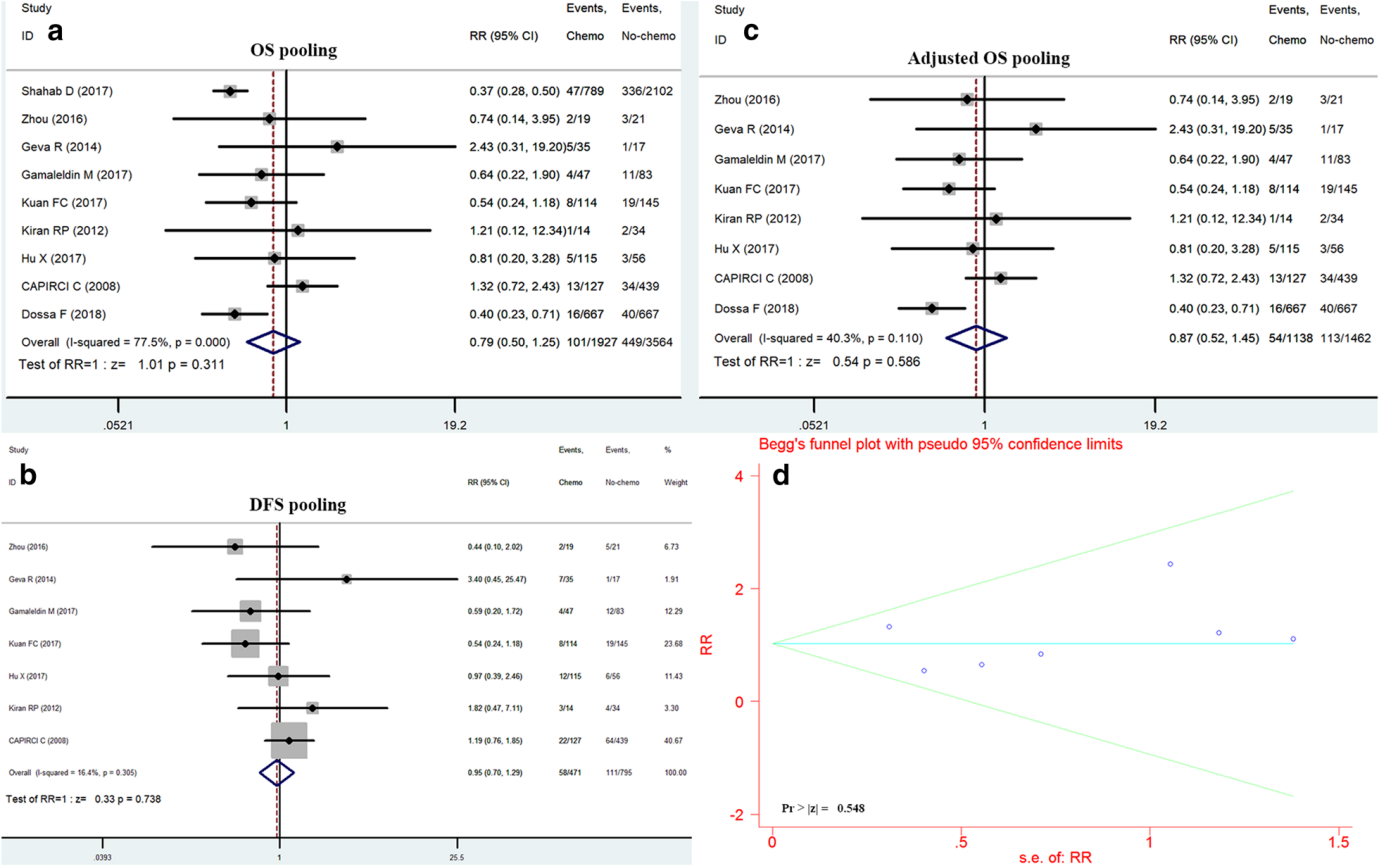
Systematic comparison of adjuvant chemotherapy after achievement of pCR. Forest plots displaying the RR with 95% confidence interval derived from the comparison of patients treated with adjuvant chemotherapy and not treated with adjuvant chemotherapy (reference group) for the pCR. **a** OS, **b** DFS, **c** Sensitivity assessments after baseline adjustment, **d** Begg’s funnel plot for publication bias

Regarding to 5-year overall survival, the pooling data did not produce a statistically significant effect on survival prognosis in cases of adjuvant chemotherapy performed, RR = 0.79, 95% CI 0.5–1.25, p = 0.311, while heterogeneity was significant, I^2^ = 77.5% (see Fig. 6a). In addition, five-year DFS were also available for this comparison. Consistently, adjuvant chemotherapy similarly did not reduce the risk of disease progression (RR, 0.95; 95% CI 0.7–1.29; p = 0.738; Fig. 6b). what is more, no statistical heterogeneity was detected, I^2^ = 16.4%.

To identify potential sources of heterogeneity, sensitivity evaluations were carried out and further to examine the influence of different inclusion standards on the overall survival estimate. A subgroup analysis suggested that adjuvant chemotherapy consistently did not affect the 5-year overall survival (RR = 0.87, 95% CI 0.52–1.45, p = 0.586, no heterogeneity observed, I^2^ = 40.3%, see Fig. 6c), when baseline demographics characteristics balanced.

To test publication bias in the present study, visual inspection of Begg’s funnel plot identified symmetry (Fig. 6d) and the Egger linear regression test and the Begg rank correlation test also revealed no evidence of bias (p = 0.548).

## Discussion

To our knowledge, this study is the first and largest study among Chinese population assessing the association between pCR and long-term prognosis. There are two main complementary findings highlighted in this study: 1) No association between improved OS and DFS and the reception of adjuvant chemotherapy after achievement of pCR; Meanwhile, the pretreatment factors of advanced T stage, positive EMVI and CRM remained no associated with worse outcomes in the context of a pCR. and 2) cumulative incidence of distant relapse surged rapidly on the 12 months and rose up to peak in the 36th month.

This current study is unique in comparing adjuvant chemotherapy against observation alone in context of pCR. In fact, previously there also existed some small sample size trials or sub-group analysis concerning to the role of adjuvant chemotherapy for patients with pCR. Consistently, those who received adjuvant chemotherapy had no survival benefits compared to those without adjuvant chemotherapy on rectal cancers with pCR [8]. Similarly, even during a median follow-up of 57 months, the 5y-DFS rates for patients in the chemo group and the no-chemo group were also comparable, showing no significant difference [9]. However, the retrospective design nature, small size sample and selection bias of those studies frequently were considered to be the potential drawbacks, hence, no consensus was arrived on the basis of pCR in determining the necessity for adjuvant chemotherapy.

Here a largest baseline balanced cohort demonstrated adjuvant chemotherapy had no overall and disease-free survival profit in pCR, which added more clear evidence on the no benefit of chemotherapy applied in pCR rectal cancers. The main reason was lied to accurate estimation for post-operative pathology, as final pathologic stage is most prognostic of oncologic outcomes in locally advanced rectal cancer patients after neo-adjuvant chemoradiotherapy [19]. It can be determined that most predictive of overall and disease-free survival was based on final pathologic T classification and N classification elements, while this applicability to the present pCR population in question is direct. Moreover, besides tumor response to preoperative therapy and final pathologic stage as strong predictors of survival, oncological outcome is most influenced by preoperative T stage and high-risk factors, such as EMVI and CRM [20, 21]. Then we stratified patients according to the clinical covariates, and identified the impact of adjuvant chemotherapy on survival in different subgroups. Similarly, there were no differences in survival with respect to positive for EMVI and CRM or not, no matter Mono or Combined Chemotherapy was administered. In addition, chemotherapy may be more effective in patients with poor prognosis, such as those with stage III or stage II with T3-4, just as the ADORE trial examining the role of oxaliplatin and leucovorin as chemotherapy for rectal cancer, it turned out that patients with final pathological stage III received improving survival but patients with stage I and II disease did not [22]. That is why we did not observe any survival profit in pCR when adding chemotherapy. Additionally, achievement of pCR seemed to have an excellent prognosis regardless of utilization of adjuvant chemotherapy, or the potential benefit of chemotherapy in patients with a pCR after neo-chemoradiation would appear to be small if it exists at all. The only way to prove the absence of such a small benefit must require a large sample size trial. That is why our study has been carried out here, as this pooled analysis covered not only 5491 patients with pCR in the whole world, but also the carefully followed patients at our single center. In total, the sample size more than 4000 pCR was enough to reach a power of consolidated conclusion, while, the benefit of adjuvant chemotherapy cannot be detected on the patients with pCR.

However, against our study, a potential advantage providing from adjuvant chemotherapy was demonstrated in three retrospective studies [14, 18, 23]. In fact, those studies were all based on the National Cancer Database (NCDB). Although the patients sample seemed very large, while, the surgical procedure was not clarified sufficiently, just demonstrating Partial proctectomy or Total proctectomy. However, others studies including our FUSCC data, which did not observe benefit of adjuvant chemotherapy in pCR, all performed TME. That may be the major reason accounting for the discrepancy. In another word, those patients from NCDB with pCR might undergo local resection, instead of TME, devoid of reginal lymph nodes resection. In addition, another explanation can be attributed to the fact that report only presented overall survival rather than DFS in their analyses, which allowed not cancer-related comorbidities to affect survival outcomes. Given that the tumor behavior is routinely evaluated by DFS, DFS is one of the most sensitive factors of the intended effects of biological characteristic. Unlike overall survival, DFS is less influenced by disparities in the treatment of relapse disease, management of comorbidity, and differential rates of death from competing causes irrelative to cancer. Additionally, the retrospective design and unbalanced baseline of that study may be considered to be other additional potential reasons.

Generally, approximately 80% of disease recurrences after radical surgery of colorectal cancer occurred within the first 2 years post-surgery [24, 25]. Nevertheless, it does not appear to be the case for achievement of pCR in our study. It inferred from some studies that about 80% of the recurrences occur within the first 4 years, which was postponed in patients with pCR [26]. That parallels the findings here, as among the cases developing distant disease relapse, relapse remained occur more than 3 years after the surgery. We therefore recommended the perspective that achievement of pCR require more prolonged close follow care [27].

Owing to insufficient data, we were unable to perform a subanalysis for different basic characters or concomitances, such as the lymph nodes retrieved and clinical TNM staging, which had important implication in the fates of outcome. Elsely, reporting bias is inherent in any retrospective database, so appropriate adjustment for potential confounders is performed to validate the effect of adjuvant chemotherapy. Although no heterogeneity in the pooling analysis was found in the present analysis, the results of a large-scale, dedicated, randomized controlled trials are awaited to determine the external validity of our findings.

## Conclusion

Rectal cancer patients with pCR did not benefit from adjuvant chemotherapy after preoperative chemoradiotherapy and radical surgery, which supports the results of previous studies investigating the role of adjuvant chemotherapy in yield pathological stage. The value of adjuvant chemotherapy for pCR patients should be further determined in prospective randomized trials or large multicenter cohort studies, in addition, we recommended that achievement of pCR require more prolonged close follow care in case of distant metastasis.

## Data Availability

All the data used to support the findings of this study are included within the article. Please contact author for data requests.

## Abbreviations

CRT: neo-adjuvant chemoradiotherapy
CRC: colorectal cancer
pCR: pathology completed response
FUSCC: Fudan University Shanghai Cancer Center
OS: overall survival
DFS: disease-free survival
CRM: circumferential resection margin
EMVI: extramural venous invasion
